# Comparative Analysis of Morbidity and Mortality Outcomes in Elderly and Nonelderly Patients Undergoing Elective TEVAR: A Systematic Review and Meta-Analysis

**DOI:** 10.3390/jcm12155001

**Published:** 2023-07-29

**Authors:** Angelos Frisiras, Emmanuel Giannas, Stergios Bobotis, Ilektra Kanella, Arian Arjomandi Rad, Alessandro Viviano, Kyriakos Spiliopoulos, Dimitrios E. Magouliotis, Thanos Athanasiou

**Affiliations:** 1 Faculty of Medicine, Imperial College London, Charing Cross Hospital, London W6 8RF, UK; angelos.frisiras18@imperial.ac.uk (A.F.); emmanouil.giannas18@imperial.ac.uk (E.G.); stergios.bobotis18@imperial.ac.uk (S.B.); ilektra.kanella18@imperial.ac.uk (I.K.); 2 Medical Sciences Division, University of Oxford, Oxford OX1 3AZ, UK; arian.arjomandirad@gmail.com; 3 Department of Cardiothoracic Surgery, Imperial College NHS Trust, Hammersmith Hospital, London W12 0HS, UK; alessandro.viviano@nhs.net; 4 Department of Cardiothoracic Surgery, University of Thessaly, Biopolis, 41 110 Larissa, Greece; spiliopoulos@uth.gr; 5 Unit of Quality Improvement, Department of Cardiothoracic Surgery, University of Thessaly, Biopolis, 41 110 Larissa, Greece; dimitrios.magouliotis.18@alumni.ucl.ac.uk; 6 Department of Surgery and Cancer, Imperial College London, St Mary’s Hospital, London W2 1NY, UK

**Keywords:** TEVAR, thoracic aorta, endovascular repair, elderly, age, morbidity, mortality

## Abstract

Objective: Due to an ever-increasing ageing population and limited available data around the use of thoracic endovascular aortic repair (TEVAR) in elderly patients, investigating its efficacy and safety in this age cohort is of vital importance. We thus reviewed the existing literature on this topic to assess the feasibility of TEVAR in elderly patients with severe thoracic aortic pathologies. Methods: We identified all original research studies that assessed TEVAR in elderly patients published up to 2023. Morbidity, as assessed by neurological and respiratory complications, endoleaks, and length of stay, was the primary endpoint. Short-term mortality and long-term survival were the secondary endpoints. The Mantel–Haenszel random and fixed effects methods were used to calculate the odds ratios for each outcome. Further sensitivity and subgroup analyses were performed to validate the outcomes. Results: Twelve original studies that evaluated elective TEVAR outcomes in elderly patients were identified. Seven studies directly compared the use of TEVAR between an older and a younger patient group. Apart from a shorter hospital stay in older patients, no statistically significant difference between the morbidity outcomes of the two different cohorts was found. Short-term mortality and long-term survival results favoured the younger population. Conclusions: The present meta-analysis indicates that, due to a safe perioperative morbidity profile, TEVAR should not be contraindicated in patients based purely on old age. Further research using large patient registries to validate our findings in elderly patients with specific aortic pathologies and both elective and emergency procedures is necessary.

## 1. Introduction

Thoracic endovascular aortic repair (TEVAR) has achieved rapid adoption as a treatment modality for thoracic aortic disease since its introduction in 1992. The minimally invasive nature of TEVAR and its short-term safety and efficacy have led to its expanding utilization as the primary treatment approach for various aortic pathologies, including penetrating atherosclerotic ulcers and aortic transections [1]. TEVAR has been associated with decreased in-hospital mortality rates, reduced intraoperative blood loss, and perioperative morbidity compared to open surgical repair [2]. The minimally invasive nature of TEVAR and its short-term safety and efficacy have led to its increasing implementation as the mainstay of treatment for thoracic aortic aneurysms (TAAs) and thoracic aortic dissections (TADs) [3].

While there is substantial evidence supporting the use of TEVAR in nonelderly patients with a low-risk factor profile, concerns have been raised about its suboptimal outcomes in certain populations, particularly the elderly. Multiple studies have shown increased postoperative morbidity, such as stroke and pulmonary complications [4,5,6], and poor mortality outcomes [5,7] in the elderly population when compared to younger counterparts. Furthermore, increasing age has been described as an independent risk factor for higher mortality among patients undergoing TEVAR [3]. This could be attributed to the increased comorbidities, frailty, and age-related physiological changes observed in the elderly population, posing unique challenges in the management of thoracic aortic disease. Consequently, the available data on the morbidity and mortality outcomes of TEVAR in the elderly are limited due to the small number of such patients who have undergone the procedure and received a systematic follow-up.

Thus, the question of whether TEVAR should be offered to this age group remains unanswered. This is particularly important as, due to an ever-increasing ageing population, it is projected that by 2050 more than 50% of aortic dissections will occur in patients over 75 years old [8,9]. In light of the changing demographic landscape, there is an increasing need for compelling evidence to determine the efficacy of TEVAR across different age groups and whether it should be offered to the elderly population.

In this context, we designed a systematic review and meta-analysis aiming to evaluate TEVAR morbidity and mortality outcomes in elderly patients. This was achieved by comparing the morbidity and mortality outcomes of TEVAR between elderly and nonelderly patients. The primary endpoint was perioperative morbidity defined as (1) endoleaks, (2) neurological complications, (3) respiratory complications, and (4) length of hospital stay (LoS), and secondary outcomes included in-hospital mortality and long-term survival following TEVAR.

## 2. Materials and Methods

### 2.1. Literature Search

This study was conducted in accordance with a protocol agreed upon by all authors and the Preferred Reporting Items for Systematic Reviews and Meta-Analysis (PRISMA) guidelines [8]. The Medline (PubMed) literature was systematically searched for studies reporting on outcomes in elderly patients undergoing TEVAR. The last day of the search was the 4 February 2023. The following MeSH terms were used in our search: “TEVAR”, “octogenarians”, “elderly”, “age”, “nonagenarians”, “outcomes”, “complications”, and “mortality”. Articles were also identified using the function “related studies” on the PubMed platform. Duplicate articles were removed. Included studies were (1) original reports with ≥10 patients, (2) written in the English language, (3) performed on human subjects, and (4) reported on elderly patients undergoing TEVAR. Abstracts, letters to the editor, and case reports were excluded. A kappa-coefficient analysis regarding the level of agreement for the inclusion of the selected studies was performed.

### 2.2. Data Extraction

Data extraction was performed independently by two reviewers (AF and SB) into a comprehensive Excel spreadsheet. In cases of discrepancy, studies were discussed in group meetings with the senior authors (DEM and TA) until a consensus was reached. The following information was extracted from each study: first author, year of publication, study-population characteristics, study design, baseline patient characteristics, number of patients operated using TEVAR, the age limit for defining the elderly population, and postoperative outcomes.

### 2.3. Patient Characteristics

This study attempted to compare the outcomes of elective TEVAR in elderly and nonelderly patients. Studies which reported on outcomes of urgent TEVAR only and on debranching TEVAR were excluded. The majority of the studies included, however, did not distinguish or conduct subgroup analysis between elective and emergency TEVAR. The elderly patients were defined as the experimental group and nonelderly patients as the control group. From the included studies, eight studies used 80 years of age for defining elderly [4,9,10,11,12,13,14,15], three studies used 75 years of age [1,16,17], and one subgrouped patients in >75 and >80 age groups [5]. Studies that used cutoff points below the age of 75 were excluded from the analysis. 

The baseline preoperative patient characteristics of the included studies are presented in Table 1. All patients included in this study suffered from a thoracic aortic pathology which was treated using the TEVAR procedure. More specifically, these included thoracic aortic aneurysms (TAA), aortic dissections, other [penetrating aortic ulcer (PAU), traumatic aortic injury (TAI), or intramural haematoma (IMH)]. The detailed aortic pathology of patients in each study is presented in Table 2.

Seven studies directly compared TEVAR outcomes between elderly and nonelderly patients [1,4,5,10,12,14,17], with three studies comparing emergency and elective TEVAR procedures [5,9,11]. In addition, six studies conducted KM analysis investigating the overall survival (OS) in elderly patients undergoing TEVAR [1,4,5,13,15,16]. A minimum of three studies per outcome were needed for data synthesis and analysis. In cases where less than three studies reported on a complication, such as renal complications, a comparison was not possible.

### 2.4. Endpoints of Interest

#### 2.4.1. Primary Endpoint

The primary endpoint of this study was to assess and compare the perioperative morbidity of TEVAR between elderly and nonelderly patients. As mortality trends often favour younger populations, the decision to select morbidity outcomes as the primary endpoint was made. Morbidity was defined based on the following outcomes: (1) neurological complications (2), endoleaks, (3) respiratory complications, and (4) length of hospital stay. Due to the heterogeneity of definitions of neurological complications provided by the papers examined, the umbrella term ‘neurological complications’ was tilized, which involved cerebrovascular accidents (CVAs) and paralysis/paraplegia. Studies that reported separately on the aforementioned outcomes were combined and reported under ‘neurological complications’.

#### 2.4.2. Secondary Endpoint

The secondary endpoint of this study was mortality, defined as a (1) short-term mortality, specifically either 30-day or in-hospital mortality, and (2) overall survival following TEVAR procedures. Both 30-day mortality/in-hospital mortality and overall survival were compared in elderly and nonelderly patients.

Study quality and risk of bias assessment were performed using the Risk of Bias in Non-Randomized Studies of Interventions tool (ROBINS-I) to evaluate non-RCTs [19]. No RCTs reporting on outcomes in the elderly following TEVAR were identified in the literature. Two reviewers (EG and AF) rated the studies independently, and the final decisions were achieved by consensus in cases of any disagreement. 

### 2.5. Subgroup Analysis: Elective vs. Emergency

Where available, data on the outcomes of emergency and elective TEVAR were used for a sensitivity analysis to explore how the outcomes in the elderly population were affected by the nature of the operation. The only outcome in which there was sufficient data to enable such a subgroup analysis regarding emergency and elective operations was short-term mortality.

### 2.6. Statistical and Sensitivity Analysis

The Mantel–Haenszel random and fixed effects methods were used to combine the odds ratio (OR) for outcomes of interest allowing for comparison between elderly and nonelderly populations. Due to the heterogeneity of the studies’ populations and surgical techniques, a random effect analysis was necessary to account for the variance within each study and between them. For funnel plots, the fixed-effect method was chosen because their purpose is not to estimate an overall effect size but rather to visually inspect the distribution of study results and identify potential bias [20]. Two strategies were used to assess the heterogeneity of the data. Firstly, publication bias was explored graphically using funnel plots. Secondly, sensitivity analysis was performed using the leave-one-out method. This involves conducting a meta-analysis on each subgroup of studies per outcome by excluding exactly one study. 

Pooled overall survival analysis was performed using the published Kaplan–Meier graphs from the included studies using a two-stage approach [18]. In stage one, raw data coordinates, time, and survival probability were extracted for elderly patients from the Kaplan–Meier curves of 9 studies. Kpondonou et al. were excluded from OS analysis as the number of patients at risk at specific time points was not available and individual patient data could not be extracted. During stage two, individual patient data were reconstructed using data coordinates based on the raw data coordinates from the first stage and the numbers at risk at certain time points. The reconstructed individual patient data from all the included studies were then pooled and a Kaplan–Meier graph was produced. The threshold for statistical significance was set as a *p*-value of less than 0.05. Data and statistical analysis were performed using RevMan V5.4 (The Cochrane Collaboration) and IBM SPSS Statistics (Armonk, New York, United States).

## 3. Results

**Figure 1:**
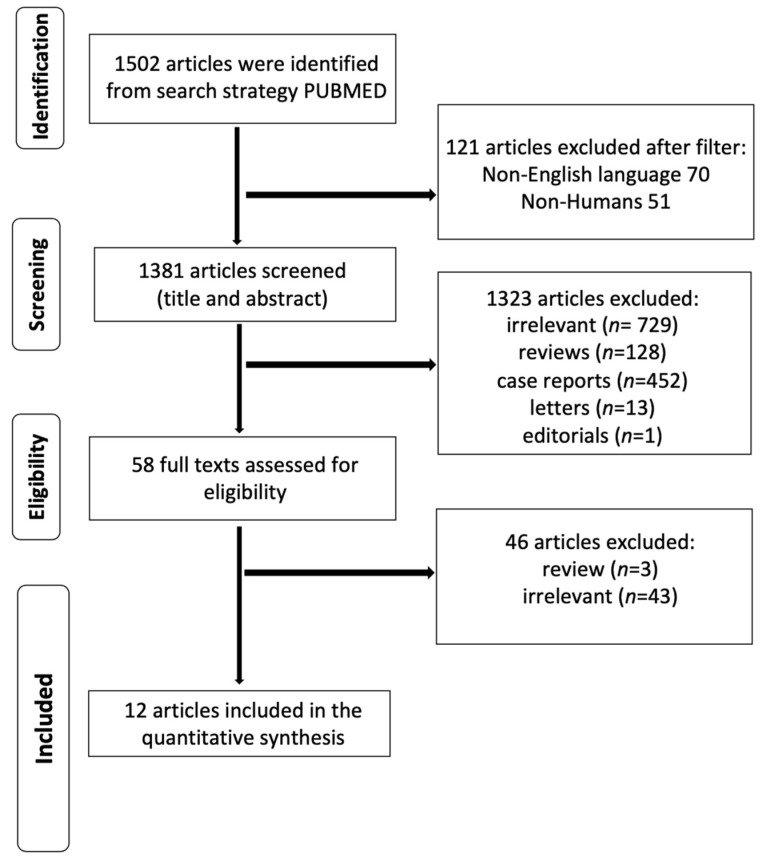
Flow Chart of the current systematic review and meta-analysis.

**Figure 2:**
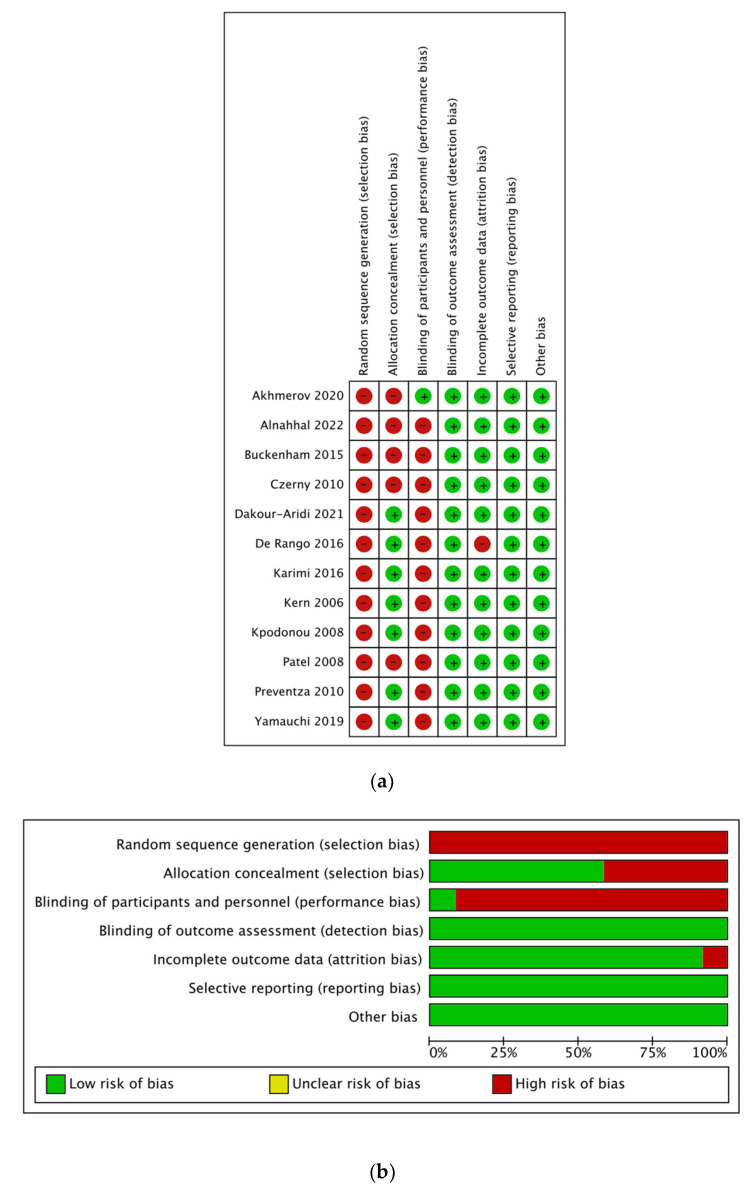
(**a**) Risk of Bias in Non-Randomized Studies of Interventions. (**b**) Risk of Bias in Non-Randomized Studies of Interventions tool with summary plot. References: [3,6,7,11,12,13,14,15,16,17,18,19].

The total number of studies included in this analysis was 12 (Figure 1). All the included studies were retrospective cohort studies, published between 2006 and 2022. The quality assessment for the included studies is demonstrated in Figure 2. The baseline preoperative patient characteristics of the included studies are presented in Table 1. The level of agreement between the two reviewers was “almost perfect” (kappa = 0.946, 95% CI:0.934, 0.958). 

### 3.1. Primary Endpoint—Morbidity

#### 3.1.1. Neurological Complications

**Figure 3:**
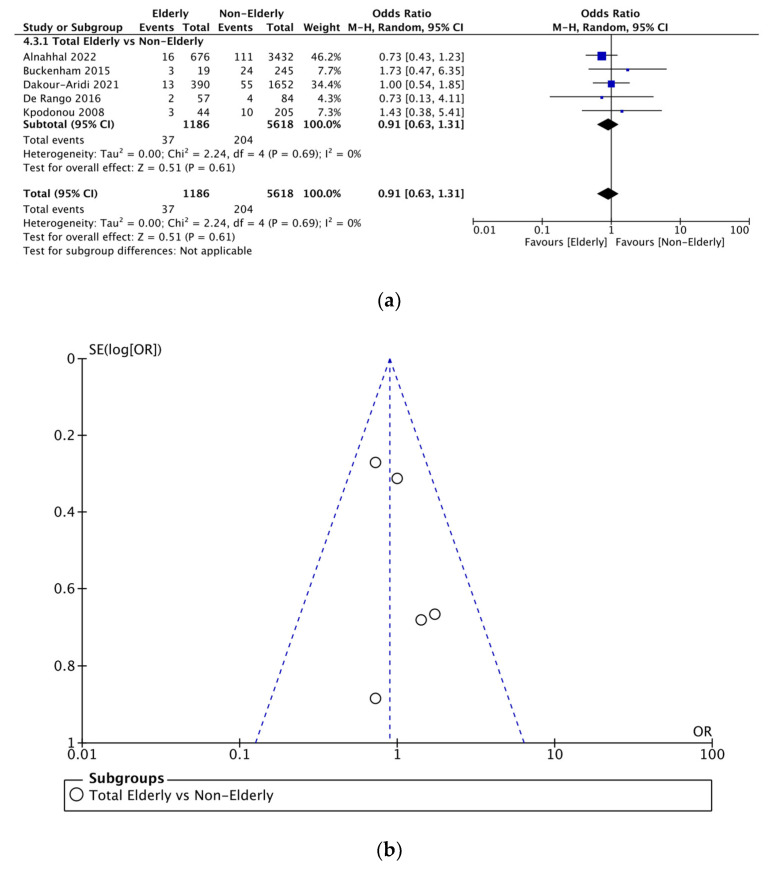
(**a**) Comparison of neurological complications between elderly and nonelderly patients undergoing TEVAR. (**b**) Funnel plot assessing the publication bias for neurological complications in elderly patients. References: [6,7,12,14,16].

Five included studies investigated the number of neurological complications between the elderly and nonelderly populations. Thus, a total of 1186 elderly and 5618 nonelderly patients were compared for this outcome. Figure 3a demonstrates that the incidence of neurological complications was not significantly different between the two cohorts (OR = 0.91 [95% CI 0.63–1.31]).

#### 3.1.2. Endoleaks

**Figure 4:**
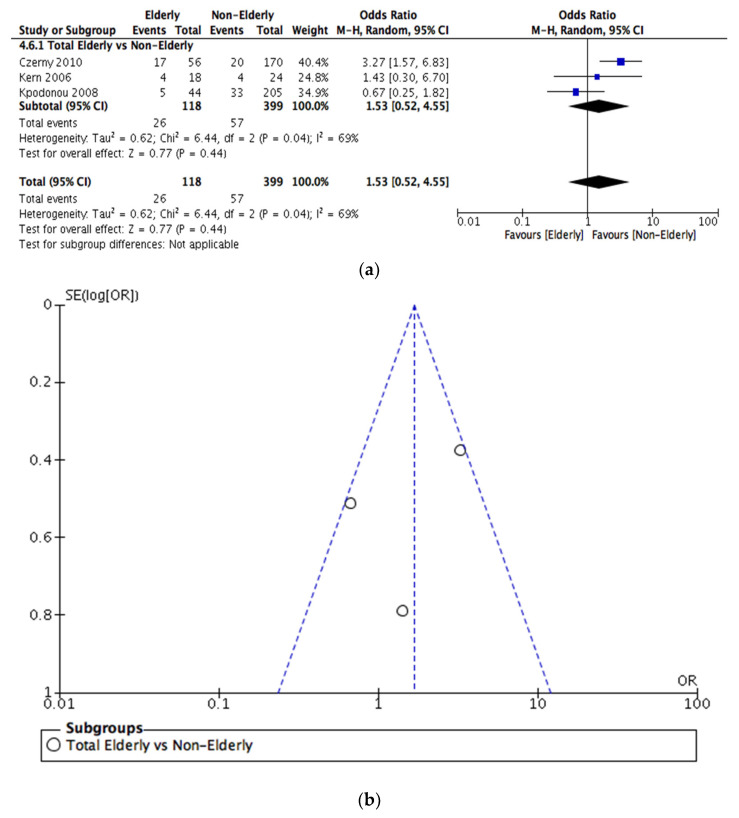
(**a**) Comparison of rate of endoleaks between elderly vs. nonelderly patients undergoing TEVAR. (**b**) Funnel plot assessing the publication bias for the rate of endoleaks. References: [3,12,18].

Endoleaks in both populations were reported in three of the included studies, amounting to a total number of 118 elderly and 399 younger patients. As shown in Figure 4a, no significant difference in the incidence of endoleaks between elderly and nonelderly populations was observed (OR = 1.53, [95% CI = 0.52–4.55]).

#### 3.1.3. Respiratory Complications

**Figure 5:**
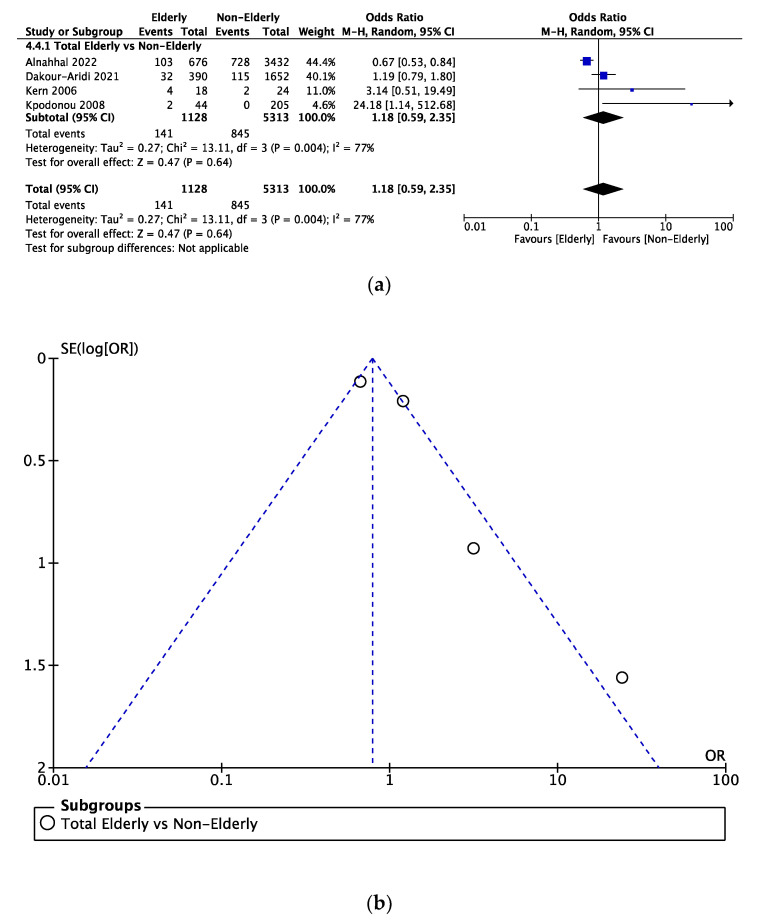
(**a**) Comparison of respiratory complications between elderly vs. nonelderly patients undergoing TEVAR. (**b**) Funnel plot assessing the publication bias for respiratory complications. References: [6,12,16,18].

Data on respiratory complications among both age cohorts were documented in four studies. This led to a comparison of 1128 elderly and 5313 nonelderly patients. There was no significant difference in the incidence of respiratory complications (OR = 1.18, 95% CI = 0.59–2.35) (Figure 5a).

### 3.2. Length of Hospital Stay

**Figure 6:**
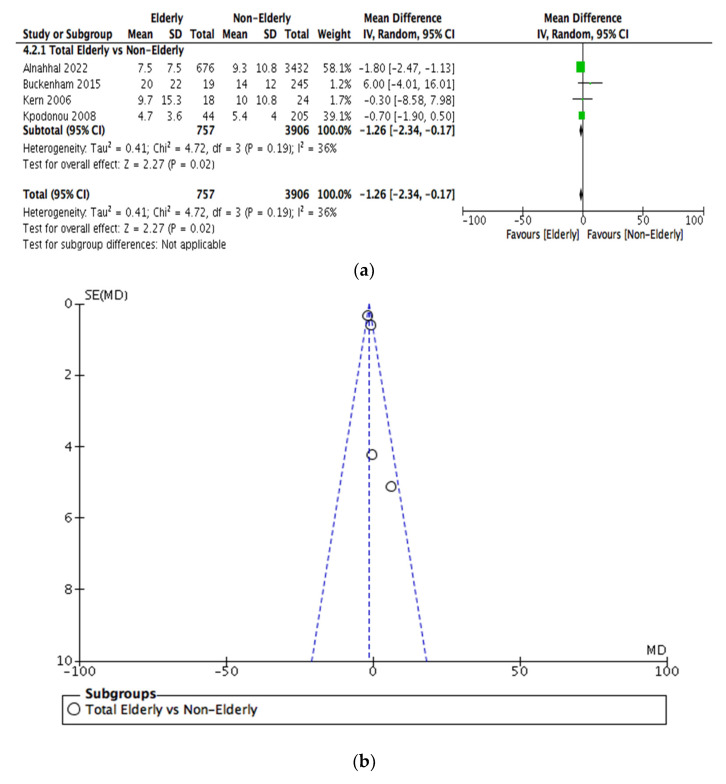
(**a**) Comparison of length of hospital stay between elderly vs. nonelderly patients undergoing TEVAR. (**b**) Funnel plot assessing the publication bias for length of hospital stay. References: [12,14,16,18].

The duration of hospital stay in both age groups was reported in four studies. Hence, 757 elderly patients and 3906 nonelderly were pooled for this outcome. Figure 6a depicts that there was a tendency towards a shorter length of hospital stay in the elderly cohort (OR = −1.26, [95% −2.34 −0.17]).

### 3.3. Secondary Endpoint

#### Short-Term Mortality

**Figure 7:**
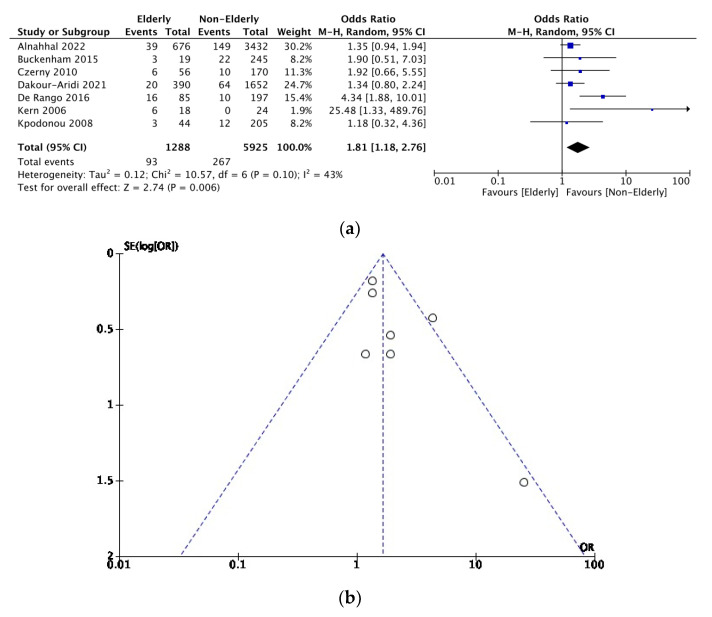
(**a**) Comparison of short-term mortality between elderly vs. nonelderly patients. (**b**) Funnel plot assessing the publication bias for short-term mortality in elderly patients. References: [3,6,7,12,14,16,18].

As this endpoint was documented in seven studies for both cohorts, a comparison of short-term mortality was carried out between 1288 elderly patients and 5925 younger patients. Figure 7a shows that short-term mortality favoured the nonelderly population compared to elderly patients (OR = 1.81, [95% CI 1.18–2.76]).

**Figure 8:**
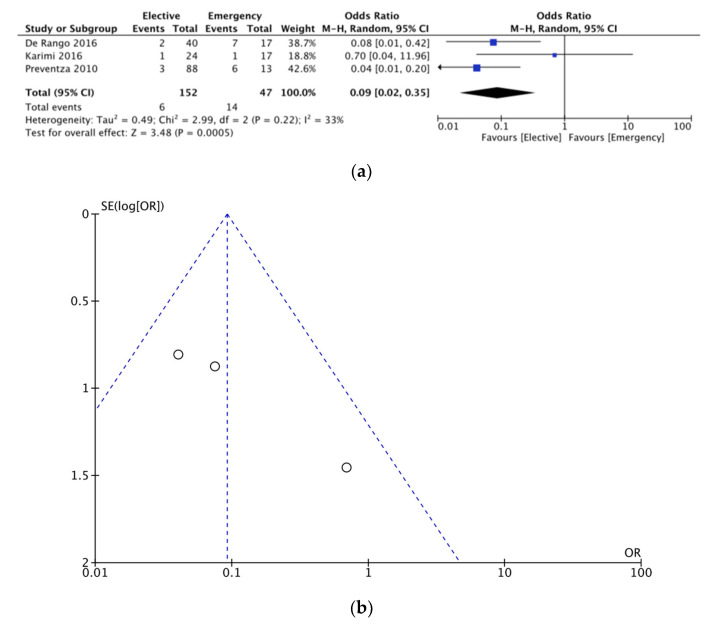
(**a**) Comparison of short-term mortality between elderly patients undergoing elective vs. emergency TEVAR procedures. (**b**) Funnel plot assessing the publication bias for short-term mortality in elective vs. emergency TEVAR procedures. References: [7,11,13].

The number of studies reporting on elective versus emergency TEVAR procedures in the elderly was three. This translated to a total of 152 patients undergoing elective operations and 47 patients undergoing emergency TEVAR. A comparison regarding short-term mortality between elective and emergency TEVAR procedures in elderly patients revealed a significantly higher mortality in emergency repairs (OR = 0.09, [95% CI 0.02–0.35]) (Figure 8a).

### 3.4. Secondary Endpoint

#### Overall Survival

**Figure 9:**
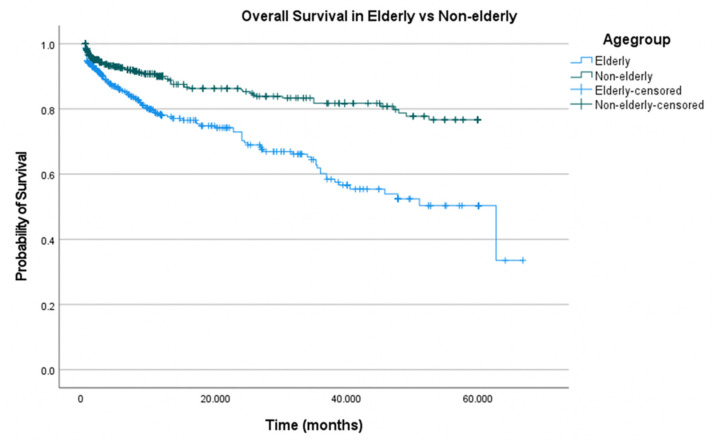
Cumulative Kaplan–Meier curve for overall survival (OS) of elderly vs. nonelderly patients undergoing TEVAR.

Figure 9 demonstrates the pooled Kaplan–Meier curves for overall survival in a cohort of 2823 patients from six studies, with median follow-up ranging from 12 to 25 months. The cohort was stratified into elderly (*n*= 633) and nonelderly (*n* = 1897) and Kaplan–Meier curves were constructed to analyse overall survival. Patients in the elderly group had a mean OS of 42.9 months compared to 50.6 months for nonelderly patients (42.9; Cl 39.7–46.2 vs. 50.6; Cl 48.9–52.3, *p* < 0.001).

### 3.5. Sensitivity Analysis

Funnel plots investigating publication bias for each outcome are presented in Figure 3, Figure 4, Figure 5, Figure 6, Figure 7 and Figure 8. The funnel plots for the incidence of neurological complications, endoleaks, and hospital stay in elderly vs. nonelderly patients (Figure 3b, Figure 4b and Figure 6b) show homogenous groups of studies. Figure 5b exhibits heterogeneity between the three studies investigating respiratory complications. Figure 7b and Figure 8b for short-term mortality resemble symmetrical inverted funnels (95% CI) with only one study being outside in the first case. 

No significant difference was found in the leave-one-out method when comparing elderly vs. nonelderly patients in the following primary endpoints: 30-day mortality, neurological complications, and respiratory complications. A significant difference was observed when excluding the studies by Kpodonou, Alnahhal, and Kern et al. [10,14,17] regarding mean hospital stay, and when excluding the study by Kpodonou et al. [10] regarding endoleaks. In the comparison of 30-day mortality between elective and urgent TEVAR, leaving out the study by Dakour-Aridi [4] also led to a statistically significant difference in the sensitivity analysis.

## 4. Discussion

This systematic review and meta-analysis demonstrated that the morbidity associated with TEVAR did not differ significantly between elderly and nonelderly patients. A shorter length of hospital stay was also identified in the older age group. Regarding our secondary endpoints, short-term mortality was found to be lower in the nonelderly population. In addition, younger patients also exhibited higher long-term overall survival rates with lower long-term mortality rates.

Neurological complications often have significant long-term health implications for patients, especially for elderly ones, and are commonly reported by studies [21,22]. Our findings did not show any statistically significant difference in post-TEVAR neurological complications between the two cohorts. Older age was also not found to be a significant predictor for stroke in a multivariable regression model of a prospective observational study [21]. Similar findings were reported by a large retrospective study investigating patients with type B dissections. The researchers’ risk analysis concluded that increasing age was not independently associated with a higher incidence of neurological complications [20]. Examples of risk factors which have been linked to higher rates of strokes and other neurological events in the existing literature include LSA coverage, prior stroke, and coronary artery disease, long duration of the procedure, aortic rupture, and female gender [20,21,22]. 

Endoleaks represent a common reason for reintervention following endovascular aortic repair, contributing to significant postoperative morbidity [23,24]. No significant difference in the rates of post-TEVAR endoleaks was found between the two age groups in this review. The lack of sufficient data distinguishing between the different types of endoleaks (I, II, or III) and documenting how long after TEVAR they were detected prevented us from carrying out further subgroup analyses. All three different types and both early and late endoleaks have been reported post-TEVAR in the literature [25,26]. Varying rates of reinterventions have been found following each endoleak type, with type II requiring the least secondary procedures [23]. A more detailed documentation of this important post-TEVAR outcome in the elderly population is, hence, needed in research studies. Nevertheless, evaluation of our results suggests no significant added risk for elderly patients undergoing TEVAR regarding the development of endoleaks.

Respiratory complications and postoperative length of hospital stay in the elderly after TEVAR are less reported in the literature. In a large US nationwide study, TEVAR was associated with decreased respiratory complications and LoS compared to open aortic repair, even though the TEVAR population was significantly older [27]. Hence, TEVAR appears to be an attractive alternative treatment option to open surgery for elderly patients, due to a lower respiratory morbidity risk. Potential reasons for the favourable LoS in the older age group after TEVAR need to be explored. Data from the endovascular repair of abdominal aortic aneurysms have shown that older age was associated with higher rates of nonhome discharge [28]. Thus, older patients who are going to be transferred to other facilities due to known comorbidities or frailty might potentially get discharged earlier than younger patients. Dissections, emergency procedures and previous cardiac history have been identified as significant predictors of prolonged hospital stay after TEVAR [29]. Considering the limited available studies for these outcomes, it is evident that more research is necessary.

Short-term mortality, either reported as 30-day or in-hospital mortality, was the endpoint most commonly documented by studies comparing post-TEVAR outcomes between elderly and younger patients (*n* = 7). Previous studies have also demonstrated that the short-term mortality of elderly patients post-TEVAR is higher compared to the nonelderly. Using data from large multinational registries, Hellgren et al. identified a higher short-term death rate in patients over 80 years old undertaking TEVAR for a variety of aortic pathologies [30]. In addition, Naazie et al. conducted a risk analysis on patients undergoing TEVAR for descending aortic aneurysms and reported that age over 75 years old is associated with higher mortality [31]. Apart from increasing age, other factors, such as female sex, coronary artery disease, ASA class, and urgency of the procedure, have also been found to be predictors of a higher early death rate [31]. Elderly patients also had lower overall survival compared to their nonelderly counterparts. Our findings regarding both short- and long-term mortality could be attributed to the generally higher prevalence of comorbidities, frailty, and cardiovascular disease risk factors among the older population [32]. However, it is important to consider the methodological limitations of the included studies, such as the absence of random treatment allocation and potential selection bias favouring elderly patients with lower comorbidities. Consequently, there is a possibility that long-term mortality rates in the elderly have been underestimated due to these limitations.

As the incidence of aortic pathologies in elderly patients continues to rise [6,7] and the applications of TEVAR for various aortic pathologies constantly expand, the evaluation of TEVAR use in older patients is becoming even more important for clinical practice. Due to its minimally invasive nature, an increasing number of patients are becoming eligible for procedural repair. Evidently, from 2003–2004 to 2011–2012, the proportion of procedural management of ruptured TAAs in patients over 80 years old performed with TEVAR increased from 18% to 86% [33]. Our analysis showed that, while increasing age is associated with higher mortality rates post-TEVAR, this trend was not observed in morbidity outcomes. With no added risk for adverse perioperative morbidity outcomes, we demonstrate that increasing age should not be a contraindication for TEVAR on its own. Quality-of-life investigations, which depict the impact of the aforementioned physical complications on patients’ overall health, are also gaining steady ground in the assessment of elderly patients. As identified by a systematic review examining thoracic aorta interventions, the postaortic surgery health-related quality of life of elderly cohorts was similar to a matched population [34]. This finding indicates a satisfactory return to the preinterventional baseline for most patients, an important consideration after every operation in older patients. Elderly patients should, hence, be considered for endovascular repair for aortic pathologies, predominantly in elective cases, unless other risk factors associated with worse TEVAR morbidity outcomes are identified. 

The population included in this review consisted of patients undergoing TEVAR for a variety of aortic pathologies. As most studies did not report separate outcome results for each diagnosis; a comprehensive analysis of how post-TEVAR morbidity and mortality varied with different aortic presentations could not be conducted. Comparisons of patient outcomes undergoing TEVAR for aortic aneurysms and dissections have yielded variable results. Although some studies report similar postoperative morbidity and mortality [35,36], inferior outcomes in patients suffering from aneurysms have also been found [37,38]. Another important consideration when assessing TEVAR use is whether the endovascular repair was carried out electively or as an emergency procedure. The lack of subgroup analyses on urgent vs. elective TEVAR in the studies included in this review meant that postoperative complications after acute and nonacute presentations could not be compared. Even for short-term mortality, the outcome most commonly reported, only three studies provided separate data for both elective and urgent aortic pathologies [5,9,11]. Death rates were lower after elective procedures, an observation generally supported by the existing literature [39,40,41,42,43]. Thus, in studies consisting of elderly populations, more detailed reporting of distinct post-TEVAR outcomes for the most common aortic presentations and for elective and emergency procedures is recommended.

The limitations of this meta-analysis greatly depend on the individual studies used, which were retrospective cohort studies, as no relevant RCTs were identified during the literature search. Due to the nature of the comparison, an RCT between the two age groups might not be feasible. Hence, future research using large TEVAR registries should be prioritised. Until then, this systematic review and meta-analysis remains the best level of evidence around TEVAR use in the elderly. In addition, as mentioned, renal complications and the effect of different aortic conditions, and the urgency of the procedure on outcomes after TEVAR, were not explored. There was also a lack of available information about other important procedural details, such as renal complications, type of anaesthesia, and duration of the TEVAR procedure. We strongly recommend that future studies report on these variables, as they can influence surgical outcomes. Patient data were derived from Kaplan–Meier curves, and not individual patient information, thus further limiting us from conducting even more subgroup analyses based on various patient characteristics. Different selection criteria between studies and confounding variables are also significant factors that could not be controlled.

## 5. Conclusions

The overall findings of this systematic review and meta-analysis suggested that morbidity following TEVAR, assessed by the incidence of neurological complications, respiratory complications, and endoleaks, was similar in elderly and nonelderly patients. Short-term mortality and long-term overall survival post-TEVAR favoured, as expected, the younger population. Due to its safe perioperative morbidity profile, it can be concluded that patients should not be excluded from elective TEVAR interventions purely based on age criteria. 

## Figures and Tables

**Table 1:** Baseline preoperative patient characteristics. Age presented as mean (±SD) or median (IQR). E: elderly, NE: nonelderly, HTN: hypertension, CVD: cerebrovascular disease, DM: diabetes mellitus, CKD: chronic kidney disease, COPD: chronic obstructive pulmonary disease, CAD: coronary artery disease, ND: nondocumented. References: [3,6,7,9,10,11,12,13,14,15,16,17].

Preoperative Baseline Patient Characteristics																				
	n		Age, yrs		Male (%)		HTN (%)		CVD (%)		DM (%)		CKD (%)		Smoking (%)		COPD (%)		CAD (%)	
	E	NE	E	NE	E	NE	E	NE	E	NE	E	NE	E	NE	E	NE	E	NE	E	NE
**Alnahhal 2022 [16]**	676	3432	83 (80–86)	64 (54–72)	333 (49)	2132 (62)	597 (88)	2707 (79)	77 (11)	315 (9)	109 (16)	531 (15)	137 (20)	600 (17)	292 (43)	137 (20)	163 (24)	708 (21)	300 (44)	901 (26)
**Dakour-Aridi 2021 [6]**	390	1652	83 (81–86)	68 (60–74)	251 (51)	984 (60)	360 (92)	1,475 (89)	42 (11)	204 (12)	63 (16)	262 (16)	186 (49)	549 (35)	231 (59)	720 (44)	123 (32)	500 (30)	74 (19)	312 (19)
**Akhmerov 2021 [17]**	25	ND	84.8 ± 3.7	ND	16 (64)	ND	24 (96)	ND	ND	ND	4 (16)	ND	ND	ND	18 (72)	ND	2 (8)	ND	ND	ND
**Yamauchi 2019 [15]**	57	ND	84.1 ± 3.4	ND	29 (51)	ND	46 (81)	ND	3 (5)	ND	8 (14)	ND	3 (5)	ND	ND	ND	8 (14)	ND	12 (21)	ND
**Buckenham 2015 [14]**	19	245	84.0 ± 3.1	59 ± 17.2	11 (58)	164 (67)	13 (68)	151 (62)	3 (16)	17 (7)	1 (5)	20 (8)	0 (0)	24 (10)	7 (37)	140 (57)	ND	ND	ND	ND
**Preventza 2010 [13]**	101	ND	83.6 ± 3.1	ND	61 (60)	ND	85 (84)	ND	2 (2)	ND	23 (23)	ND	24 (24)	ND	76 (75)	ND	33 (33)	ND	12 (12)	ND
**Kpodonou 2008 [12]**	44	205	84.0 ± 2.7	66.0 ± 11.0	27 (61)	120 (59)	34 (77)	155 (76)	1 (2)	12 (6)	ND	ND	15 (34)	45 (21)	38 (86)	150 (73)	4 (9)	40 (20)	4 (9)	17 (8)
**De Rango 2016 [7]**	57	84	67.3 ± 16.7		30 (53)	71 (85)	43 (75)	52 (62)	1 (2)	6 (7)	4 (7)	7 (8)	10 (18)	10 (12)	ND	ND	21 (37)	28 (33)	ND	ND
**Kern 2006 [18]**	18	24	78.6	62 ± 2.8	67	21	15 (83)	14(58)	5(28)	3 (13)	ND	ND	2 (11)	3 (13)	ND	ND	6 (33)	8 (33)	6 (33)	8 (33)
**Czerny 2010 [3]**	56	170	67	ND	39 (70)	53 (72)	ND	ND	ND	ND	3 (5)	13 (8)	ND	ND	ND	ND	26 (46)	51 (30)	15 (27)	39 (23)
**Patel 2008 [19]**	52	ND	80.6 ± 4.0	ND	26 (50)	ND	41 (79)	ND	ND	ND	7 (14)	ND	ND	ND	33 (64)	ND	21 (40)	ND	29 (56)	ND
**Karimi 2016 [11]**	41	ND	83.0 ± 3.0	ND	24 (59)	ND	39 (95)	ND	6 (15)	ND	7 (18)	ND	11 (27)	ND	31 (76)	ND	13 (32)	ND	8 (20)	ND

**Table 2:** Baseline preoperative aortic pathology. E: elderly, NE: nonelderly, TAA: thoracic aortic aneurysm, AD: aortic dissection, Other: penetrating aortic ulcer (PAU), traumatic aortic injury (TAI), intramural haematoma (IMH). References: [1,4,5,9,10,11,12,13,14,15,16,17].

	E			NE			Total		
Study	TAA	AD	Other	TAA	AD	Other	TAA	AD	Other
**Alnahhal 2022 [16]**	415	169	92	1328	1244	860	1743	1413	952
**Dakour-Aridi 2021 [6]**	336	54	0	1063	589	0	1399	643	0
**Akhmerov 2021 [17]**	15	7	3	N/A	N/A	N/A	15	7	3
**Yamauchi 2019 [15]**	50	6	1	N/A	N/A	N/A	50	6	1
**Buckenham 2015 [14]**	N/A	N/A	N/A	N/A	N/A	N/A	132	77	55
**Preventza 2010 [13]**	75	11	15	N/A	N/A	N/A	75	11	15
**Kpodonou 2008 [12]**	26	9	9	84	58	34	110	67	43
**De Rango 2016 [7]**	38	5	13	35	23	26	73	28	38
**Kern 2006 [18]**	N/A	N/A	N/A	N/A	N/A	N/A	N/A	N/A	N/A
**Czerny 2010 [3]**	N/A	N/A	N/A	N/A	N/A	N/A	100	57	69
**Patel 2008 [19]**	24	7	19	N/A	N/A	N/A	24	7	19
**Karimi 2016 [11]**	24	0	17	N/A	N/A	N/A	24	0	17

## Data Availability

The data that support the findings of this study are available from the corresponding author, upon reasonable request.
